# Assessment of Physiological Responses During Field Science Task Performance: Feasibility and Future Needs

**DOI:** 10.3389/fphys.2022.779873

**Published:** 2022-01-26

**Authors:** Jordan R. Hill, Barrett S. Caldwell

**Affiliations:** School of Industrial Engineering, Purdue University, West Lafayette, IN, United States

**Keywords:** human performance, spaceflight, physiological response, extravehicular activity, individual differences

## Abstract

**Objective:**

By understanding the physiological demands of different types of tasks that will be performed during extravehicular activity (EVA) on Mars, human performance safety risks can be mitigated. In addition, such understanding can assist in planning EVAs with an appropriate balance of human health and safety with scientific mission return.

**Background:**

This paper describes the results of a study of technical feasibility performed within a Mars human research analog, with participants conducting scientifically relevant planetary science sample analysis and return tasks in two distinct field locations.

**Methods:**

The authors collected heart rate, respiration rate, and heart rate variability (HRV) data, using commercial off-the-shelf hardware and software from study participants as they performed field science tasks within a concept of operations for a Mars science return human expedition mission. These data were remotely monitored, shared in real time, and later analyzed to identify different responses to different tasks in order to determine if there were any predictable or consistent patterns among participants.

**Results:**

It was ultimately determined that, while differences exist between responses to tasks, they are highly subject to multiple sources of individual variability, dynamics of evolving field science tasks, and demands of a demanding physical environment. Further, distributional analyses of participants do not support parametric statistical analysis techniques.

**Conclusion:**

The authors conclude that the physiology of individual astronauts should be extensively studied and modeled to support individualized automated monitoring tools for each crew member that is sent to Mars. Application: Physiological monitoring for specialized populations will require significant individual-level analysis, baselining, and bootstrap statistical methods to enable appropriate human performance determinations.

## Key points

This research involved remote collection and monitoring of multiple human physiological measures during field-based planetary science research and sample collection tasks. The research was conducted as part of a Mars analog research project, which involved multiple participants over multiple field deployments. Analysis of heart rate, HRV, and breathing data showed that these physiological measures could be reliably collected, monitored and shared, but could not be aggregated across or within individuals to provide simple global predictors. Parametric statistical assumptions for analysis and hypothesis testing were not supported. Thus, determining physiological health and performance zones for astronauts on long-duration planetary exploration missions are likely to require individualized, machine learning-based analysis for health status estimation.

## Introduction

To allow for human exploration of space, it is necessary to ensure that astronaut health and safety be prioritized, and that human performance capability is optimized. When astronauts arrive on Mars, many extravehicular activities (EVAs) will be expected for exploration, scientific, and life-sustaining purposes, permitting achievement of more mission objectives than could be completed only through the use of robotic landers or rovers on the surface (Newman and Barratt, 1997). EVAs, however, present considerable risk If EVAs are not developed with human health and performance in mind, low work efficiency and increased risks of EVA-related injury can result (Chappell et al., 2015). By understanding the physiological demands and physical outcomes of EVA activity, some of the associated risks can be mitigated and EVAs can be planned to reduce fatigue and improve mission outcomes. However, differences in current physical demands between EVA tasks are not currently well known in the space exploration community; this information is needed to allow for the development of EVA planning standards (Abercromby et al., 2016).

In order to address this operational gap, the authors conducted a study to demonstrate the feasibility of integrated physiological data collection, sharing, and monitoring capabilities of remote participants during the completion of field science tasks in a Mars analog research study. A number of physiological parameters were collecting using commercial off-the-shelf (COTS) hardware and software, and reported in real-time to a simulated intravehicular habitation (IVH) crew. (It should be noted that study participants were all members of the Mars analog research study, which had a primary focus on field-based planetary and biological science data collection from basaltic lava terrain settings. All participants were active members of the broader research team, and conducted other research or mission simulation tasks on a rotating basis when not participating in remote field sample collection. There was no attempt to select or standardize participants for health status; all participants were either established planetary scientists, current or former astronauts, or highly trained astronaut-like participants.) The data collected were recorded and analyzed to determine the differences in physiological responses to these EVA-like tasks, as well as to determine whether these differences were consistent or predictable across study participants.

## Background

The Martian surface is both especially hostile and fundamentally challenging to human survival and performance. There is little oxygen in the thin Martian atmosphere; the atmosphere provides little protection from harmful solar radiation, and temperatures average −60°C (−80°F) throughout the year (Dunford, n.d.; Sharp, 2012; Williams, 2016). The tasks that astronauts will be required to perform during Martian EVA will be physically demanding; performance of physical tasks may be more difficult in a reduced-gravity (0.38 that of earth) environment due to more required stabilization actions, reduced traction, and heavy spacesuits that provide protection against environmental conditions and radiation (Chappell and Klaus, 2013). These effects due to decreased gravity are compounded with space-related physiological changes: red blood cell production decreases, muscles begin to atrophy, and bone mass begins to decrease (Gunga, 2015). Historically, most injuries aboard the International Space Station occur during the performance of EVA, including contact injuries such as blisters or abrasions, or soft tissue injuries like muscle and tendon strains from overexertion (Newman and Barratt, 1997; Chappell et al., 2015).

There is a need in the space exploration community to better understand the physiological demands of EVA (Abercromby et al., 2016). Currently, the Apollo mission lunar spacewalks are the only examples of planetary EVA: not only were consumables (i.e., oxygen) depleted at rates beyond what was predicted, but measured metabolic expenditures did not map predictably to EVA phases (Miller et al., 2017). Understanding the physical demands of EVA includes the ability to identify and ensure that proper amounts of consumables (with safety margins) are included to allow for the completion of the mission, but not so much that consumable margins unnecessarily limit the available space or weight on board the spacecraft that could be allocated for other resources.

The lack of actual planetary EVA experience has necessitated the development of multiple Mars analogs by various organizations and institutions. These analogs often investigate some aspects of space exploration and are (relatively) low-risk tools to prepare for humans living and working on Mars (Rai and Kaur, 2012). NASA’s Biologic Analog Science Associated with Lava Terrains (BASALT) program is one analog that investigated scientific and operational requirements for performing scientific EVAs on Mars (Lim et al., 2019), and thus was suitable for physiological monitoring analysis. BASALT simulated EVA operational constraints in remote terrestrial environments, while conducting meaningful field science sample collection. Biological and geological sampling was performed under simulated Martian mission management conditions, including time delays on communications between a simulated Mission Support Center (MSC) and simulated Mars exploration teams. In addition, the basaltic terrains chosen for exploration are analogous to Martian composition and terrain features.

The primary focus of this study was to obtain relevant planetary science samples of interest to the research community, in addition to testing feasibility of various mission operations capabilities. BASALT differs from other Mars analogs in that the scientific samples collected during the simulated-EVA are selected and used by geologists, biologists, and other scientists in their actual planetary science research. Therefore, the collection of scientifically relevant and appropriate samples during EVA was realistically critical for these field science communities. All participants in field-based data collection were established planetary scientist researchers, current or former astronauts, or highly trained individuals with astronaut-like profiles who were trained to appropriate sample collection procedures by these researchers (a more detailed description of the BASALT research project can be found in Lim et al., 2019). The purpose of the research reported in this paper, therefore, was to determine whether there are significant differences between the physiological responses to these EVA-like tasks, and whether those differences can be quantified and used in the planning of EVA. The research presented here was a component of the overall BASALT program, but distinct from the field sample collection or evaluation of mission coordination strategies under time delay.

Several physiological parameters have been determined to be the most effective to monitor to ensure the health and safety of those living and working in remote, extreme environments, and thus were considered for inclusion in this study. Those measures included: pulse oximetry, heart rate, respiratory rate, blood pressure, core body temperature, body accelerations, stress level [usually assessed via measured heart rate variability (HRV)] and vigilance (Cermack, 2012). These measures are likely parameters that will be incorporated into physiological monitors for future Mars missions based on previous studies. In contrast to the other parameters, vigilance is not easy to measure directly, objectively, or quantitatively. Assessment of vigilance requires real-time input from the EV crewmember, and represents a secondary task intrusion into EVA task activities. Thus, vigilance was not directly assessed in this study.

Because of the physically challenging environments in which BASALT field science was conducted, it was important that the physiological monitors worn be small and not interfere with the completion of EVA tasks. (This requirement of unobtrusive, non-obstructing, and non-inhibiting physiological measurement techniques and technologies was considered an essential element of limiting additional risk to participants in a truly hostile and dangerous physical environment without on-site trauma care.) Estimates of core body temperature, while useful for ensuring the well-being of an individual in an extreme environment, would not significantly respond to changes in task in a healthy adult (although it is useful to monitor to ensure crewmember safety during the simulated EVA). It was additionally determined that kinematics (x-y-z accelerations) would be descriptive of the task itself but might not give distinctly relevant information on the physiological response to the task. These measures were deemed useful, but not in themselves critical, for determining EVA activity and EV crewmember status.

Initial evaluations to determine a commercial, off-the-shelf (COTS) monitor to use (circa 2015) indicated that few were small and unobtrusive that also measured pulse oximetry and blood pressure. The number of sensors on the EV crewmember was limited to one integrated device, to ensure that the measurement of physiological parameters did not overly inconvenience the EV crewmember or distract from task performance. It was therefore determined that heart rate, respiration rate, and HRV would be used to examine physiological changes to tasks. Heart rate and respiration rate have been used to monitor physical wellbeing in surgical settings since before the 1950s (Himmelstein and Scheiner, 1952; National Bureau of Standards, 1954) and were included in the physiological readings taken in early spaceflight, such as on the Mercury missions (Douglas, 1961). Respiration rate, in particular, has been shown to be one of the best physiological markers of physical exertion and responds most readily to changes in work rate while also being sensitive to cognitive load and other psychological stressors (Massaroni et al., 2019; Nicolò et al., 2020). HRV, derived from heart rate readings, has also been shown to decrease with increased physical and mental stress (Porges and Byrne, 1992; Braun et al., 1999; Buijs, 2013). More recently, multiple authors have considered the potential value of additional processing of HRV data, including multiple entropy or other statistical measures, as providing capabilities to distinguish more critical health indicators, such as risk of arrhythmias (Liu et al., 2013; Dong, 2016; Karmakar et al., 2017). This makes these physiological parameters good choices in exploring the physiological responses to EVA-like tasks.

Among the multiple COTS products considered for BASALT, the Zephyr BioHarness™ was selected as the product for this application (Zephyr, 2012). The BioHarness™ is worn on a strap around the chest and measures a variety of parameters at a resolution of 1 Hz, including the three parameters that were chosen for this study. The placement of the device on the chest also satisfied the need for the device to be unobtrusive and not interfere with the performance of tasks during the simulated EVA. Despite the difficulties associated with measuring physiological parameters from a moving subject in the field using an integrated, chest-worn device, e.g., see Massaroni et al. (2020)—the authors determined that a chest-worn device such as the BioHarness™ would provide more accurate data on a larger variety of parameters than a wrist-worn device without introducing obstructions to the performance of tasks in the field. The device also stores physiological readings for post-EVA download and later statistical analysis. Hill et al. (2019) provides a more detailed account of the BioHarness™ selection and integration into the BASALT architecture.

An individual’s heart rate, respiration rate, and HRV ranges are dependent on many factors. These physiological parameters can be affected by age, sex, fitness level, and/or genetic factors (Kostis et al., 1982; De Meersman, 1992; Agelink et al., 2001; Almeida and Araújo, 2003; Carter et al., 2003; Harms, 2006; Cornelissen et al., 2010) which makes defining a general, practically significant change in one of these parameters impossible without having a qualified healthcare professional take into account a specific person’s physiology. Nominal (prior to travel to the BASALT field sites) baseline physiological data for study participants was not available, as such data were not part of original BASALT participation or scientific team membership selection criteria. As described in “Materials and Methods” section below, we did try to collect imperfect baseline measurements from crewmembers during periods of inactivity for comparison, but we were not able to collect “true” baselines from crewmembers. Further, all study participants were involved in other aspects of the BASALT research program on a rotating basis when they were not conducting field-based sample collection tasks, limiting comparisons between field exposure and true rest baselines. The authors are not qualified to determine practical differences in physiological responses or define action limits for crewmembers; no such general value is available in the literature; and no specifications of physiological ranges or limits were provided by any research participant as exclusionary criteria for participation. Therefore, no practical difference thresholds or action limits were used for predictive or field data collection activity abort purposes in this study. One question within the scope of the research, however, was to determine how such action limits might be quantitatively determined from a general population or a set of specific mission candidates.

## Materials and Methods

The data were collected over the course of three BASALT deployments: one at the Craters of the Moon National Monument in Idaho (June 2016) and the other two in Hawai’i Volcanoes National Park on Hawai’i (November 2016 and November 2017). BASALT simulated the general personnel structure of the EVA by sending two extravehicular (EV) crewmembers out into the field to perform the scientific sampling while two crewmembers remain at operationally simulated instrumented habitat workstations as intravehicular (IV) crewmembers. These EV crewmembers could communicate without delay with IV crewmembers who represented co-located astronauts on the Martian surface. All communications between the EV/IV crew and MSC (who provided scientific and task management support) were delayed each way (5 or 15 min) to simulate the time it would take for communication signals to travel between Earth and Mars.

All EV and IV crewmembers were scientific participants in the BASALT research studies, with professional and scientific training in planetary science, mission operations, and/or Mars analog research settings. Additional MSC crewmembers included further planetary science, technical operations, and technical infrastructure research and development specialists. Therefore, there was a variety of participant physical fitness and capabilities to engage in mission tasks (including hiking across rough basaltic terrains, conducting visual and instrument-based sample candidate evaluations, and actual sample collections). The BASALT participants included actual astronauts and astronaut-like participants, as well as researchers not representative of likely astronauts. Because of small sample sizes (8 total participants, rotating between EV and IV roles) and the collection of very specific physiological data, additional data about participant demographics or which participants are associated with which BASALT deployments (Idaho vs. Hawai’i) would be too easily associated with specific individuals. Therefore, in keeping with data privacy and human subjects participation requirements, presentations of such data are necessarily limited in this paper.

At the beginning of each mission day, EV crewmembers would don the BioHarness™ before leaving for the field sample collection site where the simulated EVAs were performed. (It is important to note that both vehicle transport and hiking across rough terrain were required to access each sample collection site.) Throughout the simulated EVA, the BioHarness™ would record the physiological readings of each crewmember as they performed the tasks throughout the day. At the completion of each mission day, the data would be downloaded, stored, and then deleted off the devices. Each device would then recharge overnight to be ready for use during the subsequent EVA.

After the completion of the first BASALT deployment, analysis of communications and other mission data records was used to determine that crewmembers performed five different kinds of tasks. A description of each of those tasks can be found in Table 1.

**TABLE 1:** BASALT EVA tasks (Hill, 2019).

Task abbrev.	Task name	Task description
ET	Translation	Crewmembers translating within the EVA environment (walking, climbing, etc.)
EO	Observation	Crewmembers observe the EVA environment and provide those observations to the MSC (photography, vocal descriptions, contextual video, etc.)
EI	Instrument use	Crewmembers use handheld instruments to determine the geological composition of possible sampling locations.
BR	Breaking rocks	One crewmember wields a rock hammer and breaks smaller samples off a larger, desired sampling location.
BS	Bagging samples/biological sterilization	While one crewmember breaks rocks, the other dons gloves, sterilizes with alcohol, and collects the samples and puts them into numbered, cataloged sample bags.

In addition to the tasks in Table 1, baseline measurements (“Base”) were taken when convenient for participants for comparison (participants had many demands on their time). These baseline measurements were collected for two or three approximately 30-min periods when crewmembers were sitting during the Idaho deployment, or in the car to the field site before each EVA during the Hawaii deployment (approximately 10 min). The baselines were collected to allow the comparison of measurements from crewmembers during EVA to periods of relatively low stress and activity (when compared to activities performed in the field).

Throughout the completion of the simulated EVA, time stamps were taken by one of the authors (JRH) using a Microsoft Excel spreadsheet to record when tasks were being completed and for how long. JRH was able to watch the live stream of the simulated EVA to assess when crewmembers transitioned from task to task. After the completion of data collection, each point in each data set was marked with the task being performed at that point in time during the simulated EVA. Data collected that were not associated with an EVA task (during EVA prep, or during the drive home from the field site, for example) were not considered for analysis.

Associated with each BioHarness™ reading is a heart rate confidence (HR Confidence) value between 0 and 100%. The HR Confidence measure, based on an algorithmic assessment of sensor data patterns, is an indication of how confident the device is that the correct heart rate value is being recorded (100% indicates perfect certainty). At the time of publication, the BioHarness™ algorithm used the HR Confidence value as a confidence rating of the entire system (Zephyr Technology, 2016) and therefore this value was used as a quality check on all the physiological data; heart rate, respiration rate, and HRV readings with an associated HR Confidence values below 50% were not considered in the statistical analysis, to minimize inclusion of spurious data in the analysis.

Due to the frequency (1 Hz, for multiple hours, for several days’ field deployments) with which the data was sampled, the data sets for each physiological parameter were not independent. To weaken the possible influence of distributional, longitudinal dependence of the data, the mean response was taken for each occurrence of each task (i.e., each time a study participant used handheld instruments for a period of time would result in one mean response for that task). All analyses described below were performed on the mean responses to each task occurrence (instead of on the raw, dependent data).

Once data were time stamped, all low-confidence readings were eliminated from analysis, and task means were calculated. Then, Kolmogorov-Smirnov tests were conducted on the data sets of participants who participated in more than one deployment. This analysis was conducted to test whether data sets from the same participant in different locations followed the same distribution and could be combined to increase the number of data points (and statistical power) for each participant performing each task. In cases where the difference between the responses in various locations were not shown to be statistically significant, the data sets were combined into a single “case”. When there were significant differences identified between data sets for the same individual for different locations, the data were not combined, and the analyses were performed on each data set individually (e.g., Participant 1’s HRV data from the deployment in Idaho did not follow the same distribution as their HRV data collected in Hawaii, resulting in two separate “cases”). When comparing the data sets of participants who participated in two deployments (a single comparison between two data sets), an α-level of 0.05 was used. For participants who were involved in all 3 deployments (3 comparisons between 3 data sets) an α-level of 0.017 was used to offset the family error rate resulting from multiple comparisons.

Because data sets were not all normally distributed, a non-parametric, bootstrap, one-way, repeated measures ANOVA model was used with the data sets for each case (cases summarized in Tables 2–4), for each analyzed physiological parameter. This model generates multiple replicates with the same number of data points as the original data set. An ANOVA analysis is then run on each of the replicates and a confidence interval is generated to indicate whether the least square difference between two levels of the factor of interest is statistically different from zero. These serve as pairwise comparisons between the physiological responses to each task of interest and indicates whether the responses of participants to different tasks are statistically different. For more details on the generation of the bootstrap model and how it addresses the challenges associated with this kind of data set (see Hill and Caldwell, 2019).

**TABLE 2:** Heart rate cases (Hill, 2019).

Case	1	2	3	4	5	6	7	8	9	10
Participant	1	2	3	3	4	4	5	6	7	8
Location	ID/HI1	ID/HI1/HI2	HI2	ID/HI1	HI1	ID	HI1	HI1	HI2	HI2

**TABLE 3:** Respiration rate cases (Hill, 2019).

Case	1	2	3	4	5	6	7	8
Participant	1	2	3	4	5	6	7	8
Location	ID/HI1	ID/HI1/HI2	ID/HI1/HI2	ID/HI1	HI1	HI1	HI2	HI2

**TABLE 4:** Heart rate variability cases (Hill, 2019).

Case	1	2	3	4	5	6	7	8	9	10
Participant	1	1	2	3	4	4	5	6	7	8
Location	HI1	ID	ID/HI1/HI2	ID/HI1/HI2	HI1	ID	HI1	HI1	HI2	HI2

Because the bootstrap model does not assume a data distribution, *p*-values are not generated for each pairwise comparison. A conservative 99.98% confidence interval is provided instead in order to determine which differences in physiological responses are statistically significant (if zero falls within the generated confidence interval, then the difference is not statistically significant). A conservative confidence interval was used to offset the family error rate due the large number of comparisons for each case. All statistical analyses were generated using SAS^®^ software, Version 9.4 of the SAS System for Windows.

## Results

There were eight (8) participants in the study, including three female participants and five male participants. Due to the small number of participants and the fact that they were represented a limited, non-randomized pool of expert individuals, neither participant ages nor other demographics are disclosed (to protect participant confidentiality). Two individuals participated in all three deployments, two participated in two deployments, and the other four participated in only one deployment. The Kolmogorov-Smirnov tests indicated that some of the data sets of an individual who participated in multiple deployments could be combined; however, this ability to combine data sets was not consistent across all three physiological parameters or across all participants (e.g., one participant’s respiration rate data could be combined across multiple deployments, but not their heart rate data). For that reason, there were a different number of cases analyzed for heart rate, respiration rate, and HRV, summarized in Tables 2–4. Location codes refer to different deployments with “ID” being the Idaho 2016 deployment, “HI1” being the Hawaii 2016 deployment, and “HI2” being the Hawaii 2017 deployment.

After removing raw data points that were not associated with a specified EVA task, filtering out the low confidence readings, and taking mean responses to each task, 1,816 data points were used in the heart rate and respiration rate analyses. A total of 1,690 data points were used for HRV, as the algorithms used by the BioHarness™ requires a certain number of readings to get an accurate HRV reading; some tasks were of too short a duration to produce a within-task measurement. The number of data points for each individual case varied from 91 to 410 depending on the number of times a participant performed a simulated EVA, which parameter was being considered, and whether or not data sets from different locations could have been combined. This accounted for the 28 simulated EVAs performed over the course of the three deployments.

Because the authors did not have control over the number of times each participant performed any task (all tasks were performed as necessary to complete that day’s priorities for scientific data return for planetary science research goals), distribution of the number of means for each task is unbalanced. For tasks performed frequently (such as the traversing or observation tasks) there could be as many as 174 calculated means for one case, whereas tasks that were not performed as frequently (such as the breaking rocks or bagging samples tasks) there could be as few as 3 calculated means for one case. General determinations of when to conduct specific tasks were initially chosen prior to the start of the day’s field exploration; however, specific task execution was determined empirically, due to specific as well as unforeseen conditions and the desire to collect a range of geologically relevant and “interesting” samples. Unfortunately, there was also no mechanism for standardizing or calibrating participant’s perceived exertion or workload. Although the authors are familiar with cognitive and physical workload measures such as the NASA Task Load Index (TLX: see Hart and Staveland, 1988; Hart, 2006), such measures were not included in BASALT data collection protocols.

### Heart Rate

**FIGURE 1:**
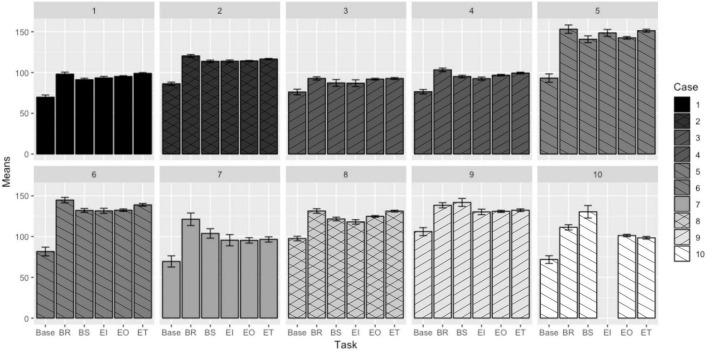
Mean heart rate responses to EVA-like tasks (Hill, 2019).

The mean heart rate responses (in beats/minute) to tasks for each case are presented in Figure 1. Each case is a separate panel in the figure; cases associated with the same participant (just during a different deployment) are shown in the same shade/with the same line pattern on the bars. The participant in the 10th case is missing a bar to indicate a mean response to the instrument use task as that participant did not perform that task at any point during the simulated EVAs in which they took part.

Pairwise comparisons were also performed between the responses to each task. The least square differences (LSDs) in responses to each pair of tasks are summarized in Table 5. The first column designates the two tasks being compared and each subsequent column presents the LSD in the mean heart rate response to each task—this is the mean heart rate response to the second designated task subtracted from the mean heart rate response of the first (i.e., Base-BR is the mean response to the breaking rocks task subtracted from the mean baseline measurement). The cells that are shaded denote differences that are not statistically significant (zero fell within the 99.98% confidence interval for the value of the LSD). The first row is to illustrate which cases are associated with the same participant.

**TABLE 5:** Differences in heart rate responses to EVA-like tasks (Hill, 2019).

Participant	1	2	3		4		5	6	7	8
							
Difference	Case 1	Case 2	Case 3	Case 4	Case 5	Case 6	Case 7	Case 8	Case 9	Case 10
Base-BR	−28.29	−34.13	−16.59	−26.86	−59.79	−63.18	−51.82	−33.82	−32.07	−39.69
Base-BS	−21.26	−27.48	−11.09	−18.71	−47.69	−50.41	−34.34	−24.12	−35.61	−58.74
Base-EI	−23.64	−27.74	−10.68	−16.09	−55.27	−50.01	−26.36	−20.48	−24.07	
Base-EO	−25.34	−28.00	−15.74	−20.37	−49.42	−50.69	−25.70	−27.25	−24.60	−29.60
Base-ET	−29.15	−30.35	−16.57	−22.90	−58.14	−57.14	−26.97	−33.63	−26.06	−26.68
BR-BS	7.03	6.65	5.50	8.15	12.10	12.77	17.48	9.70	−3.54	−19.05
BR-EI	4.64	6.39	5.91	10.77	4.52	13.17	25.45	13.34	8.00	
BR-EO	2.95	6.13	0.85	6.49	10.36	12.50	26.11	6.57	7.47	10.09
BR-ET	−0.86	3.79	0.02	3.96	1.64	6.04	24.85	0.19	6.01	13.01
BS-EI	−2.38	−0.26	0.41	2.62	−7.58	0.40	7.97	3.64	11.55	
BS-EO	−4.08	−0.52	−4.65	−1.66	−1.74	−0.27	8.63	−3.13	11.02	29.14
BS-ET	−7.89	−2.87	−5.48	−4.19	−10.46	−6.73	7.37	−9.51	9.55	32.06
EI-EO	−1.70	−0.26	−5.06	−4.28	5.85	−0.68	0.66	−6.77	−0.53	
EI-ET	−5.51	−2.61	−5.89	−6.81	−2.87	−7.14	−0.60	−13.15	−1.99	
EO-ET	−3.81	−2.34	−0.84	−2.53	−8.72	−6.46	−1.26	−6.38	−1.46	2.92

### Respiration Rate

**FIGURE 2:**
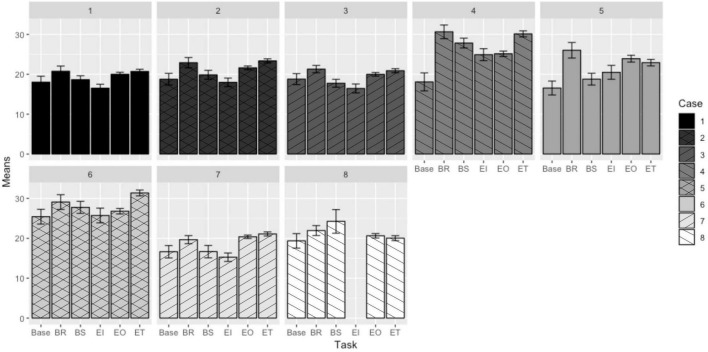
Mean respiration rate responses to EVA-like tasks (Hill, 2019).

The plot demonstrating the mean respiration rate response (in breaths/minute) by participant to each task is shown in Figure 2.

Using the same analysis methods as for the heart rate comparisons, pairwise comparisons for LSDs for respiration rate were generated and are presented in Table 6. As with the heart rate results, those non-significant differences are shaded.

**TABLE 6:** Differences in respiration rate responses to EVA-like tasks (Hill, 2019).

Participant	1	2	3	4	5	6	7	8
**Difference**	**Case 1**	**Case 2**	**Case 3**	**Case 4**	**Case 5**	**Case 6**	**Case 7**	**Case 8**

Base-BR	−2.78	−4.22	−2.49	−12.52	−9.45	−3.58	−3.10	−2.58
Base-BS	−0.65	−1.17	1.07	−9.69	−2.15	−2.39	−0.06	−4.91
Base-EI	1.46	0.74	2.35	−6.73	−3.96	−0.26	1.33	
Base-EO	−2.01	−2.88	−1.18	−7.01	−7.33	−1.31	−3.75	−1.26
Base-ET	−2.74	−4.64	−2.10	−11.92	−6.36	−5.91	−4.49	−0.65
BR-BS	2.13	3.05	3.56	2.83	7.30	1.19	3.04	−2.33
BR-EI	4.24	4.95	4.84	5.79	5.49	3.32	4.43	
BR-EO	0.77	1.34	1.31	5.51	2.12	2.27	−0.65	1.32
BR-ET	0.03	−0.42	0.39	0.60	3.09	−2.33	−1.39	1.93
BS-EI	2.11	1.91	1.28	2.95	−1.81	2.13	1.39	
BS-EO	−1.36	−1.71	−2.24	2.67	−5.18	1.08	−3.69	3.65
BS-ET	−2.09	−3.47	−3.16	−2.23	−4.21	−3.52	−4.43	4.26
EI-EO	−3.47	−3.61	−3.53	−0.28	−3.37	−1.05	−5.08	
EI-ET	−4.21	−5.37	−4.45	−5.19	−2.40	−5.65	−5.82	
EO-ET	−0.73	−1.76	−0.92	−4.90	0.98	−4.60	−0.73	0.61

### Heart Rate Variability

**FIGURE 3:**
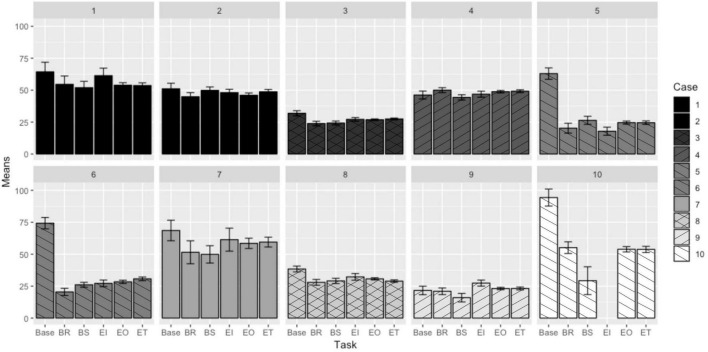
Mean heart rate variability responses to EVA-like tasks (Hill, 2019).

The plot demonstrating the mean HRV response (in milliseconds) to each task is shown in Figure 3. (Note that this study was intended to determine the feasibility of collecting reliable HRV measures in a remote field condition, not to characterize or distinguish various post-collection HRV entropy measures, such as those addressed in Karmakar et al., 2017).

Pairwise HRV task comparisons were analyzed using the same analysis as the heart rate and respiration rate comparisons. Differences between the task HRV responses are presented in Table 7. Non-significant differences are shaded.

**TABLE 7:** Differences in heart rate variability responses to EVA-like tasks (Hill, 2019).

Participant	1		2	3	4		5	6	7	8
Difference	Case 1	Case 2	Case 3	Case 4	Case 5	Case 6	Case 7	Case 8	Case 9	Case 10
Base-BR	9.71	6.25	8.11	−3.79	42.42	53.94	16.54	10.38	0.55	38.63
Base-BS	12.46	1.21	7.53	1.80	36.31	48.30	18.22	9.20	5.56	64.49
Base-EI	2.64	3.08	4.87	−0.57	44.85	47.31	6.82	6.07	−5.76	
Base-EO	10.49	5.14	5.12	−2.57	38.19	46.01	9.48	7.63	−1.63	39.80
Base-ET	10.72	2.32	4.48	−3.05	38.18	43.65	8.54	9.37	−1.70	39.90
BR-BS	2.74	−5.03	−0.57	5.59	−6.11	−5.64	1.68	−1.18	5.01	25.86
BR-EI	−7.07	−3.17	−3.24	3.22	2.43	−6.63	−9.71	−4.31	−6.31	
BR-EO	0.77	−1.11	−2.99	1.21	−4.23	−7.93	−7.05	−2.75	−2.18	1.17
BR-ET	1.00	−3.93	−3.62	0.73	−4.24	−10.30	−8.00	−1.01	−2.25	1.28
BS-EI	−9.81	1.86	−2.67	−2.37	8.54	−0.99	−11.39	−3.13	−11.32	
BS-EO	−1.97	3.92	−2.42	−4.38	1.88	−2.29	−8.73	−1.57	−7.20	−24.69
BS-ET	−1.74	1.11	−3.05	−4.86	1.86	−4.66	−9.68	0.17	−7.26	−24.59
EI-EO	7.84	2.06	0.25	−2.00	−6.66	−1.30	2.66	1.56	4.13	
EI-ET	8.07	−0.76	−0.38	−2.48	−6.67	−3.66	1.72	3.30	4.06	
EO-ET	0.23	−2.82	−0.63	−0.48	−0.01	−2.36	−0.95	1.74	−0.06	0.10

### Responsiveness of Parameters to Changes in Task

Table 8 summarizes the number of cases that demonstrated statistically significant differences (out of the total number of cases considered) for each pairwise comparison for each parameter. The occurrence of statistically significant differences is also presented as a percentage to account for the fact that one participant did not perform the instrument task and therefore the number of cases considered for each pairwise comparison is not constant. The final column sums the occurrences for a total number of statistically significant differences detected and can give an indication of the pairs of tasks most likely to demonstrate different physiological responses, and those least likely to demonstrate different responses (whether in general or by specific parameter). Due to the number of comparisons and the confidence interval used to assess statistical significance of pairwise comparisons, the family error rate of the table is 7.78%.

**TABLE 8:** Number of statistically significant differences detected per parameter (Hill, 2019).

Difference	HR	RR	HRV	Total
Base-BR	10/10 (100%)	2/8 (25%)	7/10 (58%)	19/28 (68%)
Base-BS	10/10 (100%)	1/8 (13%)	8/10 (67%)	19/28 (68%)
Base-EI	9/9 (100%)	2/7 (29%)	4/9 (36%)	15/25 (60%)
Base-EO	10/10 (100%)	4/8 (50%)	6/10 (50%)	20/28 (71%)
Base-ET	10/10 (100%)	5/8 (63%)	6/10 (50%)	21/28 (75%)
BR-BS	2/10 (20%)	2/8 (25%)	3/10 (25%)	7/28 (25%)
BR-EI	3/9 (33%)	3/7 (43%)	1/9 (9%)	7/25 (28%)
BR-EO	3/10 (30%)	0/8 (0%)	2/10 (17%)	5/28 (18%)
BR-ET	2/10 (20%)	0/8 (0%)	3/10 (25%)	5/28 (18%)
BS-EI	0/9 (0%)	0/7 (0%)	4/9 (36%)	4/25 (16%)
BS-EO	2/10 (20%)	1/8 (13%)	3/10 (25%)	6/28 (21%)
BS-ET	5/10 (50%)	1/8 (13%)	3/10 (25%)	9/28 (32%)
EI-EO	1/9 (11%)	4/7 (57%)	2/9 (18%)	7/25 (28%)
EI-ET	3/9 (33%)	6/7 (86%)	3/9 (27%)	12/25 (48%)
EO-ET	1/10 (10%)	2/8 (25%)	0/10 (0%)	3/28 (11%)
**Total**	**71/145 (49%)**	**33/115 (29%)**	**30/145 (21%)**	**134/405 (33%)**

## Discussion

Of the three physiological parameters analyzed, the heart rate readings follow the most consistent patterns across cases and appear to respond more readily to changes in EVA task, especially compared to baseline measures prior to initial traverse activity. Although there is a wide range of heart rates recorded across participants and tasks, the differences seen in responses to tasks seem to be similar, despite some inconsistencies. (For example, some participants may have had a higher heart rate response to one task over another, and other participants may have seen the reverse, due to differences in task performance and recovery demands). There are two major exceptions to the consistency of responses, and those are shown in Case 9 and Case 10. Both these cases show the bagging samples activity as eliciting a higher heart rate response than both the breaking rocks and traversing tasks, despite considerations that other participant responses (and common sense) would identify those tasks as being more physically strenuous. The bagging samples task included additional steps, as well as the consideration that improper sterilization and bagging techniques could render a sample contaminated and thus unsuitable for additional scientific analysis. As a result, it is possible (and some study participants did informally indicate) that these participants felt more mental stress when performing the bagging sample tasks, or that (since timestamps were taken on a minute scale) the occurrences of those tasks for those participants were performed after more strenuous activities.

The respiration rate responses are less consistent than the heart rate responses to the dynamics of different tasks. It does appear, however, that respiration rate did not distinguish between deployment locations, in part due to the lack of consistency within respiration rate. For respiration rate, no data sets corresponding to an individual showed statistical differences between locations (and could therefore be combined). The respiration rate pairwise comparisons also demonstrate more instances where two tasks do not elicit significantly different respiration rate responses. This could indicate that respiration rate responds less readily to changes in task, or that this was a select group of participants, or a result of relative measurement insensitivity: the authors were not able to measure tidal volume (the amount of air taken in with each breath), which also increases with the onset of physical work (Watson, 1974). Physical (not respiration-related) movements of crewmembers may also have confounded some of the respiration rate readings in the field (Massaroni et al., 2020).

As with heart rate responses, breaking rocks and traversing tasks generally elicited the highest respiration rate responses. Case 7 and Case 8 are exceptions to these trends. These cases correspond to the same participants who deviated from identified patterns when considering heart rate responses to tasks. This reinforces the idea that these individuals experienced the tasks differently, or that these tasks were performed directly after more strenuous tasks, which affected the readings taken in subsequent tasks.

Interestingly, in the case of HRV, cases that represent the same participant (Cases 1 and 2, and Cases 5 and 6) demonstrate some consistency in the responses, though there is little consistency in responses when comparing between participants. This could indicate that HRV responses are much more highly dependent on the individual instead of the task being performed, or that the individual participant experienced similar levels of stress throughout the execution of each task which were different from other participants’ stress levels but consistent across different locations. This theorized individualized stress response is also supported by the fact that there are no two pairwise task comparisons that demonstrate statistically significant differences in HRV response across all cases. Recent literature also provides evidence that HRV could be a psychophysiological index of an individual’s general resilience (An et al., 2020), and that one or more measures of statistical entropy in HRV may help identify health risks for some individuals (Liu et al., 2013; Dong, 2016; Karmakar et al., 2017). This is a particularly interesting avenue for future study, particularly how these individualized responses will influence EVA task and resource planning.

For all cases, heart rate responses were lower during baseline measurements than during all other tasks; this trend was seen in half the respiration cases. Most cases also demonstrated higher HRV responses during baseline measurements (lower mental stress) than in the field. This indicates that simply being in the field—no matter how physically challenging the task being performed—does influence physiology.

Overall, the pairs of tasks most likely to demonstrate differences in physiological responses from the baseline measurements are the traversing (75%) and observation tasks (71%). The traversing vs. baseline difference is not unexpected as traversing was considered by the authors (and in discussions with study participants) to be one of the most physically demanding tasks. However, it is surprising that the observation task (generally consisting of standing, taking photos, and verbally describing the environment) was the task with the second most likely probability to elicit a different-than-baseline response. This may be explained by the fact that the beginning of simulated-EVAs in the BASALT deployment consisted of traversing to sites of interest, interspersed with pauses for observation. The responses collected and labeled as “observation” tasks may have been affected by the more strenuous traversing tasks that occurred immediately before the observation.

The pair of tasks performed in the field that are more likely to demonstrate a difference in physiological response are the instrument use and traversing tasks (48%). This is not unexpected as the instrument use task would be considered by the authors to be one of the least physically demanding tasks performed in the field.

All field tasks compared to baseline measurements were more likely elicit statistically different physiological responses (60–75% of comparisons demonstrating statistical significance) than comparing a field task with another field task (11–48%). The pairs of tasks least likely to demonstrate differences in physiological responses are the observation and traversing tasks (11%), the bagging samples/biological sterilization and instrument use tasks (16%). Biological sterilization/bagging samples and instrument tasks demand similar levels of physical activity (stationary standing), and therefore it is also expected that the physiological responses to these tasks would not differ significantly.

One unexpected finding was that the observation and traversing tasks would not commonly elicit statistically different responses; the authors’ experience at the BASALT sites suggested traversing to be much more strenuous than observation tasks. This result may be explained by the pattern of traversing interspersed with observations at the start of each simulated-EVA (discussed above). It is also possible that some portions of observation tasks were timestamped as traversing tasks (and vice versa) due to the limitations as to the frequency with which the authors could assign timestamps (on a minute-scale), the lack of clear delineation as to when one task ended and another began, and any offsets between the internal BioHarness™ timestamp and the time on the computer on which the task timestamps were recorded.

This study identifies only statistically significant differences within individuals and between responses to tasks; there are no conclusions drawn as to whether the detected differences between physiological responses for specific individuals are practically significant. Because physiological measurements and the range of those measurements vary from person to person due to a variety of different factors, a practically significant difference would need to be determined by a qualified healthcare professional who is familiar with each individual’s physiology over an extensive period of data collection and individual analysis. While the authors can guess that differences detected on the order of 1 beat per minute of heart rate (as an example) are not practically useful, this cannot be stated with certainty, and a practical threshold cannot be determined by the authors.

The concept of determining individualized thresholds and action limits based on each participant’s activity patterns (and evaluations by medical professionals or software-based machine learning algorithms) is further reinforced by the inconsistent responses between participants (especially when considering HRV responses). The range of physiological responses to different tasks, and even between the baseline measurements of individuals, presents evidence against combining data sets and trying to set action limits or recognize abnormal physiological readings based on the combined data.

Results of this study strongly indicate the possible role of machine learning tools to help with automated, individualized monitoring of astronaut physiological states during EVA. In general, vigilance monitoring of usually nominal conditions is counter-indicated as a human performance task (Parasuraman, 1987; Warm et al., 2008). However, more recent techniques for assessing the clinical value of entropy measures of physiology demonstrate the use of multiple *post hoc* statistical analyses, with one or more entropy measures having value for specific individuals (Dong, 2016; Karmakar et al., 2017). This type of analysis is more suited for machine learning (ML) techniques that can apply evolutionary algorithms to determine the best combination of measures for a specific individual, using intra-individual comparisons as training inputs. These types of ML approaches have been suggested as viable to assist in long-term health monitoring and decision support for assessing health risks for individuals (Hijazi et al., 2016; Patel et al., 2016).

This study was not intended to determine the nature of such ML approaches, so much as to assess feasibility of remote physiological monitoring and determination of general physiological thresholds to protect participant safety and health. Any automation or machine learning algorithm set to monitor physiological responses during EVA would need to be designed and trained for the individual astronaut. Since many unforeseen tasks and evolving baselines would be anticipated for a crewmember on a multi-year mission to Mars, ML tools that can even work with ambiguous data or evolving conditions would be important to develop (Yuan et al., 2020) and tune to each individual Such individualized evaluation is feasible to expect, as the astronauts who are going to be sent to Mars will be selected years before the mission occurs. This allows for many hours of physiological data to be collected on these individuals to train automation to recognize their physiological patterns and tendencies. Similar concepts for automated monitoring have already been proposed for other parameters during space operations (Kitts and Swartout, 1998; Biswas et al., 2005), including life support (Schreckenghost et al., 1998).

All data were collected in the field during a larger Mars analog research project with a variety of complex field science sample collection tasks. These real-world conditions gave the authors little control over the order and duration in which tasks were performed. Thus, compared to a clinical trial or other controlled laboratory study, these data are less systematically manipulated (and thus of limited scientific rigor compared to a laboratory-based study with controlled conditions and tasks). However, the collection of field data during the BASALT deployments represents a unique (and previously unavailable) demonstration and real time remote presentation of remote physiological data collection in an ecologically valid scenario. The field data (and scientific relevance of resulting sample collection for mission completion) collected during BASALT are more representative of the types of activities that will be conducted during an actual Martian EVA.

The authors took away many “lessons learned” from these field deployments of particular benefit for long-term health monitoring of specific, highly skilled and trained individuals. In the future, it would be beneficial to find a device that measures respiration rate, tidal volume, and blood oxygen saturation, if possible. This would allow for better estimates of changes in consumable oxygen usage, which would benefit EVA resource planning more than simply being able to determine whether tasks elicited higher physical workloads. Additionally, the authors recommend that efforts be made to record “true” participant baselines for more accurate comparison.

The collection of physiological data at a frequency of 1 Hz and the ability of the authors to assign activity timestamps on a minute scale potentially introduced more error into the study than originally anticipated, especially when considering tasks that participants switched between fairly quickly (e.g., traversing and observation, as discussed above). The speed at which timestamps were assigned was limited by both the computer’s timestamp (likely to be a certain number of seconds off from the internal timestamp of the BioHarness™) and the authors’ reaction and typing speeds. The time-keeping author required at least 30 s to note the change in task for one or both participants, mark the time in two separate logs, label the timestamp(s), and add any required notes. A suggestion for future work to help mitigate this problem would be to have to individuals assigning timestamps (either one for each crewmember or one person to keep track of the time and the other to fill in activity labels and add notes).

The results of this study are only able to identify statistically (not practically or clinically) significant differences between physiological responses during task performance. In future iterations of this work, it would be beneficial to work with medical professionals who can become familiar with study participant physiology to give a better estimate as to what constitutes a practically significant difference in physiological responses to tasks (e.g., 1 bpm difference in heart rate may be practically significant but only changes above 10 bpm are practically significant from a health and safety standpoint). Since any future live monitoring of these types of data will be to ensure crewmember safety, these practical differences will be more useful and hold more meaning than statistical differences.

It is very difficult to simulate all aspects of Mars activity with enough fidelity to know exactly how astronauts will respond physiologically during Martian EVA, but it can be assumed that the data produced during actual Martian EVA will be subject to similar challenges of data consistency, continuity, and quality. It is important that before humans are sent to Mars, methods are put in place to handle and gain useful information from relatively “messy” data sets. This is especially crucial if such data are used for high-consequence decisions (such as termination of an EVA or initiation of emergency response procedures). Of course, physiological monitoring of human behavior in a partial-gravity setting would be extremely difficult to configure in an earthbound EVA configuration that also achieved scientifically relevant and valid sample return mission objectives. The authors acknowledge this fundamental limitation, without suggestions on how to modify analog settings such as BASALT to address those concerns.

## Conclusion

Despite imperfect data collected in multiple challenging and physically remote field settings, this study demonstrated that it was possible to deploy, collect, present, and record individual participant responses to field science tasks during simulated-EVA using COTS physiological monitoring instruments. More importantly, such data collection was able to detect statistically significant differences between and within tasks, as well as between and within individuals, across multiple hours of data collection at 1 Hz in a dangerous and remote basaltic terrain.

The authors do not draw conclusions regarding the practical significance of these differences among the field participants, due to the variability of the data and the need for medical professional expertise to determine individual action limits. The differences detected did not display consistency across participants. This may be due to individual differences in fitness, differences in environmental conditions between deployments (and days within deployments), and a lack of experimental control over task exposures.

These data do demonstrate, however, that physiological responses are highly dependent on individual participant as well as activity characteristics; therefore, it is unadvisable to simply combine data sets from multiple individuals to draw general conclusions regarding physiological performance limits. Any automation that may monitor and draw conclusions from astronaut physiological data will need to be designed to consider the individual performance patterns and experiences of each person.

Despite the inability to identify consistent differences in physiological responses to tasks, the authors can provide some “lessons learned” for others performing similar field research. The authors suggest measuring oxygen saturation and tidal volume in future research to better predict oxygen consumption, recording “true” participant baselines, assigning an additional individual to collect data timestamps (or collect data at a frequency more closely aligned with the pace at which individuals can realistically assign timestamps), and working with medical professionals to determine practical differences in physiological response. The authors also believe that studies such as this provide an important link from existing clinical assessments of established physiological monitoring parameters to new capabilities for studying real tasks and individual responses in “free range” (unconstrained) and even remote field settings (see Patel et al., 2016).

It is also important to note that only three physiological parameters—heart rate, HRV, and respiration rate—were considered in this analysis. The analysis of those parameters with traditional parametric statistical methods is not an effective way of characterizing EVA-like tasks. Other physiological parameters, including those available with commercial COTS sensors such as the BioHarness™ wearable technology, could possibly also demonstrate individual differences in responses to different tasks worthy of future investigation. Detecting statistical differences in physiological responses promises the opportunity for machine learning algorithms to be used and trained to detect individual task and physiological stress differences. If such algorithms could be developed, trained to an individual’s data, and be able to recognize task-specific or abnormal physiological readings and alert operators based on relevant physiological action limits, increased astronaut safety during EVA would be considerably enhance. Particularly with small crew sizes, assistance from software agents functioning as supplemental crewmembers would also be useful to support future Martian EVA without overloading attentional and vigilance resources by Mars-based crewmembers.

## Data Availability Statement

The raw data supporting the conclusions of this article will be made available by the authors, without undue reservation.

## Ethics Statement

The studies involving human participants were reviewed and approved by the Purdue University Institutional Review Board. The patients/participants provided their written informed consent to participate in this study. Written informed consent was obtained from the individual(s) for the publication of any potentially identifiable images or data included in this article.

## Author Contributions

JH: primary data collection and cleaning, statistical analysis (in collaboration with the Purdue Statistical Consulting Service), and primary writing of journal manuscript (based on dissertation). BC: principal investigator, initial research design, selection of physiological monitoring hardware, review statistical analysis, editing, and review of dissertation and journal manuscript. Both authors contributed to the article and approved the submitted version.

## Conflict of Interest

The authors declare that the research was conducted in the absence of any commercial or financial relationships that could be construed as a potential conflict of interest.

## Publisher’s Note

All claims expressed in this article are solely those of the authors and do not necessarily represent those of their affiliated organizations, or those of the publisher, the editors and the reviewers. Any product that may be evaluated in this article, or claim that may be made by its manufacturer, is not guaranteed or endorsed by the publisher.
